# The effects of kratom *(Mitragyna speciosa)* on metabolic syndrome-related parameters: a systematic review and meta-analysis

**DOI:** 10.3389/fphar.2025.1587528

**Published:** 2025-06-18

**Authors:** Ajaree Rayanakorn, Pattarachon Apisitwittaya, Shaun Wen Huey Lee, Kantirat Yaja, Ratchanon Inpan, Mingkwan Na Takuathung, Nut Koonrungsesomboon

**Affiliations:** ^1^ Department of Pharmacology, Faculty of Medicine, Chiang Mai University, Chiang Mai, Thailand; ^2^ Clinical Research Center for Food and Herbal Product Trials and Development (CR-FAH), Faculty of Medicine, Chiang Mai University, Chiang Mai, Thailand; ^3^ School of Pharmacy, Monash University Malaysia, Bandar Sunway, Malaysia; ^4^ Office of Research Administration, Chiang Mai University, Chiang Mai, Thailand

**Keywords:** kratom, mitragynine, metabolic syndrome, cholesterol, triglyceride, meta-analysis, Mitragyna speciosa Korth

## Abstract

**Background:**

Mitragyna speciosa Korth. [Rubiaceae] is a tropical plant with opioid-like properties used for its metabolic altering effects, but data on the metabolic effects are lacking. This systematic review and meta-analysis aim to examine the effects of kratom use on parameters related to metabolic syndrome (MetS) in humans.

**Methods:**

PubMed, Web of Science, Embase, and Cochrane Library databases were searched from inception to 6 September 2024 to identify eligible analytical studies examining the association between kratom consumption and metabolic effects. The effect estimates were summarized as mean differences (MDs) with 95% confidence intervals (CIs) and pooled using random-effects models.

**Results:**

Five cross-sectional studies were included. The meta-analysis involving 1,458 adults observed significantly lower levels of serum low-density lipoprotein cholesterol (LDL-c) (MD -0.25-0.21 mmol/L; 95% CI: −0.39, −0.02), triglyceride (MD -0.17 mmol/L; 95% CI: −0.25, −0.09), while high-density lipoprotein cholesterol (HDL-c) levels increased (MD 0.07 mmol/L; 95% CI: 0.03, 0.11). Body mass index was also lower in adults using kratom when compared to the control group (MD -1.52 kg/m^2^; 95% CI: −2.81, −0.23).

**Conclusion:**

Kratom use in the adult population was associated with lower LDL-c, TG, BMI, and higher HDL-c levels. However, the total cholesterol and blood sugar lowering effects were not statistically significant. The findings suggest that kratom use may have a protective effect against certain metabolic risk factors. Further investigations particularly longitudinal studies with larger sample sizes are required to confirm these findings.

**Systematic Review Registration:**

identifier CRD42024600702

## 1 Introduction

Metabolic syndrome (MetS) refers to a group of metabolic disorders characterized by abdominal obesity, hyperglycemia, dyslipidemia, and elevated blood pressure (Levesque and Lamarche, 2008). According to the modified National Cholesterol Education Program (NCEP) Expert Panel on Detection, Evaluation, and Treatment of High Blood Cholesterol in Adults (Adult Treatment Panel III), MetS is diagnosed when three or more of the following five criteria are met: (1) waist circumference over 102 cm for men or 88 cm for women (for individuals of Asian descent, the thresholds are 90 cm for men and 80 cm for women), (2) blood pressure over 130/85 mmHg or current use of antihypertensive drugs, (3) fasting triglyceride (TG) level over 150 mg/dL (1.7 mmol/L), (4) fasting high-density lipoprotein cholesterol (HDL-c) level less than 40 mg/dL (1.03 mmol/L) for men or 50 mg/dL (1.29 mmol/L) for women and (5) fasting plasma sugar levels of 100 mg/dL (5.6 mmol/L) or higher or on treatment for elevated blood glucose (Grundy et al., 2005). Nearly 25% of the global population is estimated to have MetS (O'Neill and O'Driscoll, 2015), and its incidence continues to rise in parallel with obesity rates (Swarup, Ahmed, Grigorova and Zeltser, 2024). Evidence also suggests a strong association between MetS with chronic inflammation and oxidative stress (Buscemi et al., 2009; Rana, Nieuwdorp, Jukema and Kastelein, 2007). These conditions contribute to endothelial dysfunction and insulin resistance. Studies to date suggest that individuals with MetS face a 2-fold increased risk of cardiovascular disease (CVD) and a 5-fold increased risk of diabetes mellitus compared to those without the condition (Samson and Garber, 2014).

Currently, MetS is managed through lifestyle modifications and pharmacological interventions. These therapeutic approaches prioritize the regulation of key physiological parameters, including blood pressure, fasting blood glucose, lipid profiles, and body weight maintenance. However, the long-term adherence to strict dietary regimens and exercise protocols often presents significant challenges for patients. This limitation has sparked growing interest in alternative therapeutic approaches for managing MetS components, particularly the potential application of *Mitragyna speciosa.*

Mitragyna speciosa Korth. [Rubiaceae] (Kratom) is a psychoactive plant of the Rubiaceae family native to Southeast Asia. Its leaves have been traditionally utilized as a herbal medicine for centuries across various forms, including fresh/dried leaves, teas, extracts, and decoctions (Hassan et al., 2013; Prozialeck et al., 2019). The plant’s active alkaloids primarily *mitragynine* and *7-hydroxymitragynine* are known to exhibit diverse activities, including modulating opioid receptors and exerting dose-dependent narcotic effects (Ahmad et al., 2022; Babu, McCurdy and Boyer, 2008). Medicinally, kratom has been used in treating multiple medical conditions including symptoms of opioid withdrawal syndrome, pain, insomnia, anxiety, cough, fever, hypertension, diabetes, dyslipidemia, and enhancing work performance (Ahmad et al., 2022; Assanangkornchai, Muekthong, Sam-angsri, and Pattanasattayawong, 2007; Boyer, Babu, Adkins, McCurdy and Halpern, 2008; Gong, Gu, Xu and Kang, 2012; Hassan et al., 2013; La-Up, Wongrith, Chaichan, Aramrattana and Saengow, 2022; Prozialeck et al., 2019; Veltri and Grundmann, 2019). However, the evidence concerning kratom’s health benefits remains inconclusive with inconsistent findings across studies.

Recent research on kratom’s effects on weight loss has shown promising results in both animal and human models (Chittrakarn, Sawangjaroen, Prasettho, Janchawee and Keawpradub, 2008; La-up et al., 2021; La-Up et al., 2022). However, its impact on lipid profiles appears to be variable, as previous studies have suggested the potential negative impact on metabolic health among opioid users with findings of increased serum triglyceride (TG), low-density lipoprotein cholesterol (LDL-c) levels, and systolic blood pressure (SBP), alongside decreased high-density lipoprotein cholesterol (HDL-c) levels (Aghadavoudi, Eizadi-Mood and Najarzadegan, 2015; Rahimi, Gozashti, Najafipour, Shokoohi and Marefati, 2014). Given its opioid-like effects, kratom has also been linked to adverse health effects including cardiovascular diseases, atherosclerosis, and hypertension (Leong Bin Abdullah and Singh, 2021; Trakulsrichai et al., 2015). Despite the widespread use of kratom, the risks and benefits of kratom remain poorly investigated. To date, no research has comprehensively examined the long-term impact of kratom on serum metabolic profiles. This systematic review and meta-analysis aimed to examine the effects of kratom consumption on metabolic parameters relating to MetS in humans.

## 2 Methods

This study was conducted in accordance with the Preferred Reporting Items for Systematic Reviews and Meta-analyses (PRISMA) statement (Moher, Liberati, Tetzlaff, Altman and The, 2009; Page et al., 2021). The study protocol was registered at the International Prospective Register of Systematic Reviews (PROSPERO: CRD42024600702). The study was granted exemption by the Research Ethics Committee of the Faculty of Medicine, Chiang Mai University (no. EXEMPTION 0619/2024).

### 2.1 Search strategy and study selection

A comprehensive search was conducted across four relevant databases including PubMed, Web of Science, Embase, and Cochrane Library using the following keywords: (“kratom” OR “mitragynine” OR “mitragyna” OR “Mitragyna speciosa”) AND (“metabolic syndrome” OR “metabolic” OR “lipid level” OR “dyslipidemia” OR “lipid” OR “cholesterol” OR “triglyceride” OR “lipoprotein” OR “fatty-acid” OR “FPG” OR “fasting plasma glucose” OR “HbA1C” OR “RBS” OR “random blood sugar”). The search was limited to humans, with no additional restrictions up until 6 September 2024 (Online, Supplementary Appendix SA1). Articles were included if there were: 1) primary studies comparing metabolic parameters between individuals who have used kratom compared to non-users or control group irrespective of study design, and 2) reported at least one of the primary outcomes. Studies investigating the effects of kratom and/or its derivatives for other indications, as well as those focused on other derivatives, abstracts, conference proceedings, letter to editor or laboratory experiments were excluded. Additionally, reference lists of the identified articles were reviewed to uncover other potentially eligible studies.

The primary outcomes of interest were body mass index (BMI), lipid profiles including serum triglyceride (TG), total cholesterol (TC), HDL-c, LDL-c levels, and fasting blood glucose. The secondary outcomes were blood pressure, waist circumference, body weight, and liver function test (LFT).

### 2.2 Data extraction

Studies were searched and screened by one reviewer (PA) which was reviewed and checked against inclusion criteria by another reviewer (AR). AR and PA independently performed data extraction, which was confirmed by KY. Disagreements were discussed through consultation process until a consensus was reached between reviewers and all authors.

Extracted information included first author, the year of publication, sociodemographic characteristics, kratom use characteristics (duration, average frequency, quantity), BMI, metabolic parameters, study design, the number of participants from exposure and control groups, length of kratom use, the outcomes and key findings. Data extracted were compared and mapped to the baseline measurements and outcomes at the end of follow-up to obtain differences or changes occurred during kratom exposure.

### 2.3 Risk of bias assessment

Cross-sectional studies were evaluated using a modified version of the Newcastle-Ottawa Scale (NOS) for cross-sectional studies (Modesti et al., 2016). The studies were assessed based on three broad perspectives: 1) the selection of the study group, 2) the comparability of the groups, and 3) the assessment of the outcome. A maximum star score of 5, 2 and 3 were allocated for selection, comparability, and outcome respectively resulting in a total possible score of 10. Studies scoring 8 or higher were categorized as having a low risk of bias, those scoring 6-7 were considered as having a moderate risk of bias, and scores of 5 or less indicated as having a high risk of bias (Ribeiro et al., 2020). The tool contained 12 questions according to several criteria including study objective, selection of study population and representativeness, sample size, outcome measurement, blinding of outcome assessors, statistical analysis, and follow-up rate. Each study was classified as low risk of bias when 75%–100% of responses were “yes”, moderate risk of bias when 25%–75% were “yes”, and high risk of bias when “yes” responses were 25% or less (Kunstler et al., 2018; National Heart and Blood Institute, 2021). Three reviewers (AR, PA, KY) independently assessed the risk of bias among the included studies. Any disagreements were discussed and resolved through consultation process to reach a consensus.

### 2.4 Statistical analysis

Prior to effect size estimation, all outcome measures were converted to the same units, where possible. The effect sizes were calculated by the mean difference between the exposed group (kratom users) and the non-exposure group (non-kratom users). In case the mean and standard deviation (SD) values were not reported, we calculated the mean and the standard deviation (SD) using the Wan et al. method (Wan et al., 2014). A random effect meta-analysis was conducted using the “meta” package in R-studio version 2024.12.0 (Posit Software, PBC, Boston MA), build 467, with a p-value <0.05 identified as statistically significant. The effect size for each study was reported as the mean difference (MD) with the 95% confidence intervals (CIs). Cochran’s Q test and I^2^ statistic were used to assess heterogeneity (Higgins, 2008). A subgroup analysis was also conducted based on the type of control groups (non-kratom users and low-dose kratom users with up to three kratom tea or juice glasses per day). Sensitivity analyses were executed using several estimation models, including the Dersimonian-Laird, Paule-Mandel, and Sidik-Jonkman methods, along with the exclusion of high risk of bias studies to determine the optimal component for the random-effects model.

## 3 Results

### 3.1 Study selection

**FIGURE 1:**
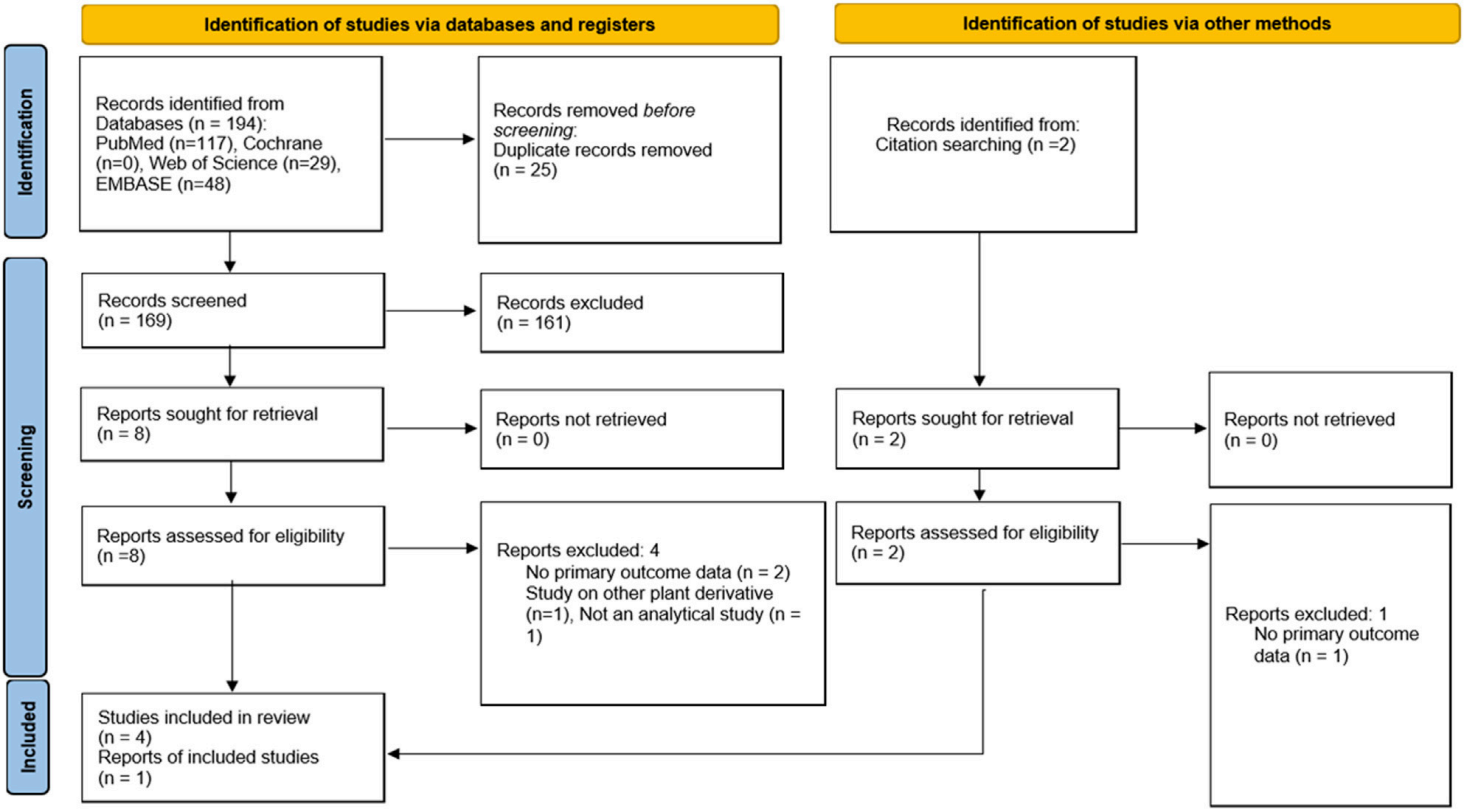
Flow diagram of search strategy and study selection.

We identified 194 publications, with 169 articles after deduplication. Following title and abstract screening, 161 articles were excluded, leaving eight articles for full-text eligibility assessment. Additional 2 records were identified through citation searching which underwent full-text assessment. Of the 10 articles, five were excluded. Three did not report any of the primary outcomes (Ahmad and Aziz, 2012; Fauzi et al., 2022; Saingam et al., 2023) while one each investigated other plant derivatives (Musa Obadia et al., 2024) or was not an analytical study (Ramachandram, Chia Siang and Rini, 2023) (Supplementary Table S1.1). Therefore, there were five studies included in the systematic review and meta-analysis (Abdullah et al., 2020; La-up et al., 2021; La-Up et al., 2022; Singh et al., 2018a; Singh et al., 2018b). The flow chart detailing the study selection process is presented in Figure 1.

### 3.2 Study characteristics

The five cross-sectional studies included, with a total of 1,458 participants (737 in the exposure group; 721 in the control group) (Abdullah et al., 2020; La-up et al., 2021; La-Up et al., 2022; Singh et al., 2018a; Singh et al., 2018b). All studies were conducted in Southeast Asia, with three studies in Malaysia (Abdullah et al., 2020; Singh et al., 2018a; Singh et al., 2018b), and two studies in Thailand (La-up et al., 2021; La-Up et al., 2022). The study durations ranged from 3–9 months. Three studies did not provide information on the study duration (La-up et al., 2021; La-Up et al., 2022; Singh et al., 2018a). Most studies included only male participants except for two studies that included both males and females (La-up et al., 2021; La-Up et al., 2022). Among these, males were predominant kratom-users whereas the majority of non-kratom users were females. The mean age of kratom users and non-kratom users ranged between 25.6–55.8 years, and 28.9–55.7 years respectively. The characteristics of the included studies are summarized in Table 1.

**TABLE 1:** Summary of study characteristics.

No	First author and year	Country	Study design	Study period	Sex	Sample size, n (%)		Mean age (years±SD)		Duration of kratom use (N)		Average daily consumption patterns of kratom			
						Kratom users (male, %)	Non-kratom users (male, %)	Kratom users	Non-kratom users	1–5 years	<5 years	Number of glasses consumed per day	Volume per serve (mL)	Kratom form	*Mitragynine* daily dose (mg)
1	Abdullah et al. (2020)	Malaysia	Cross-sectional	9 months	Male	100	100	29.7± 18.1^a^	29.7± 6.0^a^	n = 44	n = 56	4.5	350	kratom tea/juice	NR
2	La-up et al. (2021)	Thailand	Cross-sectional	NR	Mixed	285 (78.6)	296 (30.4)	55.8± 11.4	55.7± 12.0	NR	NR	NR	NR	NR	NR
3	La-up et al. (2022)	Thailand	Cross-sectional	NR	Mixed	285 (78.6)	296 (29.7)	55.8± 11.4	55.7± 12.0	NR	NR	NR	NR	NR	NR
4	Singh et al. (2018a)	Malaysia	Cross-sectional	NR	Male	58	19	25.6± 7	28.9± 5.6	n = 41	n = 59	3.5	270-360	kratom tea/juice	76.3–114.8
5	Singh et al. (2018b)	Malaysia	Cross-sectional	3 months	Male	9^b^	10^b^	30± 5.6	NA	n = 7	n = 12	3 (n = 10) >3 (n = 9)	280-320	kratom tea/juice	76.2–94.2

*Note:*

^a^ The mean and standard deviations were calculated using Wan et al. method.

^b^ The study compared outcomes between kratom users who used >3 glasses vs up to three glasses of kratom tea/juice daily; NA, not applicable; NR, not reported; SD, standard deviations.

The average kratom consumption was reported in three studies ranging between 3 and 4.5 glasses per day and more than half of kratom users had been consuming kratom for more than 5 years (Abdullah et al., 2020; Singh et al., 2018a; Singh et al., 2018b). One study recruited only kratom users and compared the effects of kratom among those who consumed more than three glasses vs up to three glasses of kratom juice/tea daily (Singh et al., 2018b). The dose of *mitragynine* was measured in two studies using Gas Chromatography–Mass Spectrometry (GC-MS) with an average of 76.3–114.8 mg and 76.2–94.2 mg of *mitragynine* daily respectively (Singh et al., 2018a; Singh et al., 2018b) (Table 1). Among low-dose kratom users (consuming up to three glasses of kratom juice/tea daily), the estimated average daily *migragynine* intake ranged from 21.8–80.7 mg, based on the *mitragynine* content per glass in the processed kratom juice samples, which varied between 21.8 and 26.9 mg (Singh et al., 2018b). The mean duration of kratom use was reported in two studies which were 82.7 months and 91.45 months respectively (Singh et al., 2018a; Singh et al., 2018b).

### 3.3 Risk of bias assessment

Among the five cross-sectional studies, two studies had a low risk of bias with a score of 9 (Abdullah et al., 2020) and 8 (La-up et al., 2021). Two studies were rated as moderate risk of bias with a score of 6 (Singh et al., 2018a; Singh et al., 2018b), while the remaining study received a score of 5 (La-Up et al., 2022), indicating a high risk of bias (Table 2). The main reasons for high risk of bias were attributable to no description of sampling strategy, justification of sample size and response rates, and the use of non-validated measurement tool (La-Up et al., 2022).

**TABLE 2:** Results of risk of bias assessment for cross-sectional studies using the Newcastle-Ottawa Scale.

Cross sectional studies	Selection				Comparability		Outcomes		Total (maximum 10)
	Representativeness (★)	Sample size (★)	Non-respondents (★)	Ascertainment of the exposure (risk factor) (★★)	The study controls for the most important factor (select one) (★)	The study control for any additional factor (★)	Assessment of outcomes (★★)	Statistical test (★)	
Abdullah et al. (2020)	★	★	—	★★	★	★	★★	★	9
La-up et al. (2021)	★	—	—	★★	★	★	★★	★	8
La-up et al. (2022)	—	—	—	★	★	★	★	★	5
Singh et al. (2018a)	★	—	—	★★	—	—	★★	★	6
Singh et al. (2018b)	★	—	—	★★	—	—	★★	★	6

### 3.4 Meta-analysis

**FIGURE 2:**
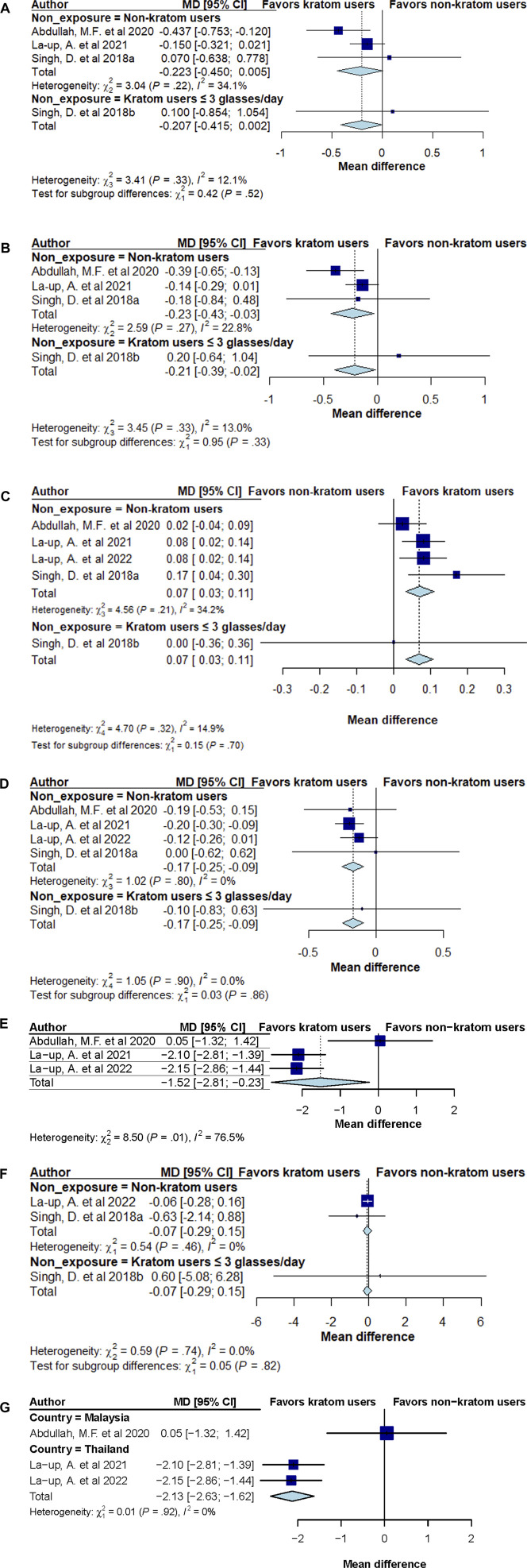
**(A)** Forest plot summary of total cholesterol mean difference (MD) and 95% confidence intervals (CIs) comparing kratom users and non-kratom users. **(B)** Forest plot summary of low-density lipoprotein cholesterol (LDL-c) mean difference (MD) and 95% confidence intervals (CIs) comparing kratom users and non-kratom users. **(C)** Forest plot summary of high-density lipoprotein cholesterol (HDL-c) mean difference (MD) and 95% confidence intervals (CIs) comparing kratom users and non-kratom users. **(D)** Forest plot summary of triglyceride mean difference (MD) and 95% confidence intervals (CIs) comparing kratom users and non-kratom users. **(E)** Forest plot summary of body mass index (BMI) mean difference (MD) and 95% confidence intervals (CIs) comparing kratom users and non-kratom users. **(F)** Forest plot summary of fasting blood sugar (FBS) mean difference (MD) and 95% confidence intervals (CIs) comparing kratom users and non-kratom users. **(G)** Forest plot summary of body mass index (BMI) mean difference (MD) and 95% confidence intervals (CIs) comparing kratom users and non-kratom users by study population’s country.

A total of five cross-sectional studies involving 1,458 participants (737, kratom users; 721, non or low-dose kratom users) which reported at least one of the primary outcomes were included in the meta-analysis (Figures 2A–G).

#### 3.4.1 Total cholesterol (TC)

The combined random-effect model results showed a trend towards a reduction in total cholesterol (TC) levels among kratom users compared to those with low or no kratom use (MD -0.207 mmol/L; 95% CI: −0.415, 0.002), with minimal evidence of heterogeneity (I^2^ = 12.1%, P = 0.33; Figure 2A). No difference was noted among participants consuming more than three glasses of kratom tea/juice daily versus those consuming up to three glasses (MD 0.100 mmol/L; 95% CI: −0.854, 1.054).

#### 3.4.2 Low-density lipoprotein cholesterol (LDL-c)

Pooled analyses showed that kratom consumption led to a greater reduction in LDL-c levels compared to control groups (MD -0.21 mmol/L; 95% CI: −0.39, −0.02), with little evidence of heterogeneity (I^2^ = 13.0%, P = 0.33; Figure 2B). Importantly kratom users was found to report significantly lower LDL-c levels compared to non-kratom users (MD -0.23 mmol/L; 95% CI: −0.43, −0.03). However, no difference was noted between individuals who had high consumption of kratom tea/juice (>3 glasses daily) compared to those with low kratom consumption (≤3 glasses) (MD 0.20 mmol/L; 95% CI: −0.64, 1.04).

#### 3.4.3 High-density lipoprotein (HDL-c)

Pooled analyses showed that kratom consumption also led to an increase in HDL-c compared to control group (MD 0.07 mmol/L; 95% CI: 0.03, 0.11), with little evidence of heterogeneity (I^2^ = 14.9%, P = 0.32; Figure 2C). Subgroup analysis among kratom users compared to non-kratom users indicated consistent findings (MD 0.07 mmol/L; 95% CI: 0.03, 0.11), with no significant difference between those with high (>3 glasses daily) vs low quantity (≤3 glasses daily) of kratom consumption (MD 0.00 mmol/L; 95% CI: −0.36, 0.36).

#### 3.4.4 Triglyceride (TG)

Pooled analysis demonstrated that kratom users had a greater reduction of TG levels compared to the control group (MD -0.17 mmol/L; 95% CI: −0.25, −0.09, Figure 2D), with little evidence of heterogeneity (I^2^ = 0.0%, P = 0.90). Subgroup analysis comparing kratom users to non-users yielded consistent results (MD -0.17 mmol/L; 95% CI: −0.25, −0.09). As observed with other outcomes, the analysis found no significant difference in TG levels between high and low kratom consumption groups (MD -0.10 mmol/L; 95% CI: −0.83, 0.63).

#### 3.4.5 Body mass index (BMI)

Meta-analysis also revealed that kratom use was associated with a significant decrease in BMI compared to healthy individuals (MD -1.52 kg/m^2^; 95% CI: −2.81, −0.23), with relatively high heterogeneity among the studies (I^2^ = 76.5%, P = 0.01, Figure 2E). The significant reduction in BMI was notably greater in two cross-sectional studies conducted in Thailand involving both male and female participants (La-up et al., 2021; La-Up et al., 2022). Subgroup analysis by study population’s country (Thailand vs Malaysia) showed that among study participants from Thailand, BMI reduction was significantly greater in kratom users compared to non-users (MD -2.13 kg/m^2^; 95% CI: −2.63, −1.62), with no heterogeneity observed between studies (I^2^ = 0%, P = 0.92). In contrast, no significant difference in BMI was observed between kratom users and non-users in the Malaysian population (MD 0.05 kg/m^2^; 95% CI: −1.32, 1.42) (Figure 2G).

#### 3.4.6 Fasting blood sugar (FBS)

Slightly lower fasting blood sugar (FBS) levels were observed among kratom users compared to control groups, but the difference was not statistically significant (MD -0.07 mmol/L; 95% CI: −0.29, 0.15). The subgroup analysis comparing kratom users vs non-kratom users yield consistent results (MD -0.07 kg/m^2^; 95% CI: −0.29, 0.15) whereas the analysis comparing individuals consuming >3 glasses of kratom tea/juice vs those consuming up to three glasses found no significant difference in FBS levels (MD 0.60 kg/m^2^; 95% CI: −5.08, 6.28). No significant heterogeneity was observed between studies (I^2^ = 0.0%, P = 0.74, Figure 2F).

### 3.5 Sensitivity analyses

Sensitivity analyses employing different models including Paule-Mandel, DerSimonian-Laird, Maximum-likelihood, Hunter-Schmidt found that results of primary outcomes were mostly similar, even after excluding studies with a high risk of bias (Supplementary Table S3.1–3.6).

## 4 Discussion

To our knowledge, this is the first systematic review and meta-analysis examining the effects of kratom on metabolic syndrome-related parameters in humans. The meta-analysis revealed a significant reduction in serum LDL-c, TG levels, and BMI, along with a significant increase in HDL-c level among kratom users as compared to the control group. A favorable trend toward lower TC and FBS levels was observed in the kratom-consuming group compared to those with no or low consumption although the difference was not statistically significant. A comparison of kratom users consuming more than three glasses versus up to three glasses of kratom tea or juice daily showed no statistically significant differences in any outcome measures. This suggests no clear dose-response relationship between kratom intake and lipid profiles, where higher consumption does not necessarily lead to additional reductions in lipid levels. However, limited sample size might have influenced non-significant findings.

Several mechanisms might contribute to kratom’s potential lipid-lowering effects. The primary alkaloid, *mitragynine* exerts its efficacy largely through agonist activity at mu and kappa opioid receptors, which are involved in regulating various physiological processes including lipid metabolism (Czyzyk et al., 2010). An *in vitro* study suggested that *mitragynine* may stimulate the lipolysis pathway via α2b- and β1-adrenergic receptors, leading to the activation of adenylyl cyclase (AC) and subsequently inducing lipolysis process (Khoirul Rista et al., 2022). In addition, the anti-inflammatory effects of *mitragynine* through the inhibition of cyclooxygenase-1 (COX-1) and COX-2 expression, resulting in prostaglandin E2 (PGE2) suppression (Utar et al., 2011) may indirectly contribute to improved lipid profiles and help regulate HDL-c balance. In the present work, the beneficial effects of kratom on lipid profiles was observed in the primary analysis, except for TC. However, a significant reduction in TC observed in some estimation models including Paule-Mandel, DerSimonian-Laird, and Maximum-likelihood suggests its potential benefits in lowering TC levels. Small sample size and potential confounding factors including lifestyle, dietary behaviour, demographic difference, and co-existing conditions may have influenced the results.

Kratom has also demonstrated antioxidant and antidiabetic properties. According to an animal model, kratom extract significantly inhibited α-glucosidase enzyme activity, improved hyperglycaemia, dyslipidaemia, oxidative stress and hepatorenal alternations, and reduced body weight gain in diabetic rats (Zhang et al., 2023). In our meta-analysis, kratom consumption was associated with a significant lower BMI despite significant heterogeneity. The variation in the study population may have contributed to the observed clinical heterogeneity. Notably, one study was conducted in Malaysia among young male participants (Abdullah et al., 2020), whereas the other two studies took place in Thailand and included both male and female participants with a mean age of 56 (La-up et al., 2021; La-Up et al., 2022). The more pronounced BMI reduction observed in the two studies conducted in Thailand may have been attributable to the inclusion of older population, who are generally more likely be obese or overweight compared to younger individuals.

According to the findings of this meta-analysis, the blood sugar lowering effects among kratom users was not statistically significantly. It is important to note that the pooled analysis results were based on only three studies comprising 677 individuals, one of which had a high risk of bias. Although, a trend toward lower FBS was observed, the evidence remains inconclusive and may be influenced by sample size, study population, and potential methodological flaws in the included studies.

This systematic review and meta-analysis provides valuable insight into the clinical implications of long-term kratom use on metabolic parameters in humans. Despite the paucity of evidence, the present study poses some strengths. Firstly, we performed comprehensive searching in several databases with no time or language restrictions as well as citations searching to identify potential studies evaluating the effects of kratom on humans’ metabolic profiles. Secondly, a subgroup analysis by the type of control group was conducted to reduce bias related to the inequality of control groups. Thereby, the control groups were divided into two categories, namely, non-kratom users and low dose kratom users with up to three glasses of kratom tea or juice intake daily. Thirdly, a highly accurate search and explicit inclusion and exclusion criteria resulted in inclusion of relevant articles into the analysis. Fourthly, a rigorous quality assessment and sensitivity analysis were done to ensure the robustness of the results. However, there are some limitations to be noted. Firstly, the findings were based on observational data with potential for confounding effects and selection bias. Additionally, one study did not mention about the sampling strategy (Singh et al., 2018b) while the others employed non-random sampling strategy (Abdullah et al., 2020; La-Up et al., 2022; Singh et al., 2018a), further limiting the generalizability of the findings. The variability in the methodology and risk of bias of the included studies, particularly the differences in control group types posed challenges in determining whether the data were combinable, especially for BMI. However, we accounted for this potential clinical heterogeneity by conducting subgroup meta-analysis based on control group type and found no evidence of heterogeneity between studies. Secondly, variations in kratom preparation, alkaloid concentrations, and individual metabolic responses might have led to different levels of exposure across studies and influenced the results. The legal status and regulatory restrictions of kratom across different settings can hinder the enforcement of quality control standards, contributing to variability in product preparation and limiting comparability across studies. Thirdly, the limited number of studies and sample size may reduce the accuracy of effect estimates and culminate in non-significant findings. Therefore, larger and more rigorous studies with standardized kratom preparation and well-defined control groups are needed to confirm kratom’s potential benefits in human metabolic profiles. Fourthly, heterogeneity across studies which might be attributable to study population, methodology, intervention differences may impact the certainty of the study findings. However, our meta-analysis results showed no or low heterogeneity for all primary outcomes except BMI. Fifthly, most participants in the studies included were males which may not represent gender characteristics and biological differences. Nevertheless, this was consistent with the fact that the majority of kratom users are males. Finally, all the studies included were conducted in Southeast Asia countries, which may limit generalizability of the findings to other regions as they may not reflect the characteristics of different populations. Therefore, the analysis results should be interpreted with caution.

## 5 Conclusion

This systematic review and meta-analysis suggests that kratom consumption is associated with lower serum LDL-c, TG, BMI, and higher HDL-c levels in adult populations. A trend toward lower total cholesterol levels was observed, reaching statistically significance in certain estimation models. The findings indicate that kratom use may have a protective effect against certain metabolic risk factors, potentially reducing the risk of MetS and development of CVD. However, it is important to note that the analysis is based on observational studies and cannot establish causality. Further research, particularly longitudinal studies with standardized kratom preparation, larger sample size and well-defined control groups are warranted to confirm these associations.

## Data Availability

The original contributions presented in the study are included in the article/Supplementary Material, further inquiries can be directed to the corresponding author.

## References

[B1] Abdullah M. F. I. L. B. Tan K. L. Isa S. M. Yusoff N. S. Chear N. J. Y. Singh D. (2020). Lipid profile of regular kratom (Mitragyna speciosa Korth.) users in the community setting. PLoS ONE 15 (6), e0234639. 10.1371/journal.pone.0234639 32525924 PMC7289408

[B2] Aghadavoudi O. Eizadi-Mood N. Najarzadegan M. R. (2015). Comparing cardiovascular factors in opium abusers and non-users candidate for coronary artery bypass graft surgery. Adv. Biomed. Res. 4 (1), 12. 10.4103/2277-9175.148294 25625118 PMC4300596

[B3] Ahmad I. Prabowo W. C. Arifuddin M. Fadraersada J. Indriyanti N. Herman H. (2022). Mitragyna species as pharmacological agents: from abuse to promising pharmaceutical products. Life 12 (2), 193. 10.3390/life12020193 35207481 PMC8878704

[B4] Ahmad K. Aziz Z. (2012). Mitragyna speciosa use in the northern states of Malaysia: a cross-sectional study. J. Ethnopharmacol. 141 (1), 446–450. 10.1016/j.jep.2012.03.009 22440259

[B5] Assanangkornchai S. Muekthong A. Sam-angsri N. Pattanasattayawong U. (2007). The use of mitragynine speciosa (“Krathom”), an addictive plant, in Thailand. Subst. Use and Misuse 42 (14), 2145–2157. 10.1080/10826080701205869 18097996

[B6] Babu K. M. McCurdy C. R. Boyer E. W. (2008). Opioid receptors and legal highs: salvia divinorum and Kratom. Clin. Toxicol. 46 (2), 146–152. 10.1080/15563650701241795 18259963

[B7] Boyer E. W. Babu K. M. Adkins J. E. McCurdy C. R. Halpern J. H. (2008). Self-treatment of opioid withdrawal using kratom (Mitragynia speciosa korth). Addiction 103 (6), 1048–1050. 10.1111/j.1360-0443.2008.02209.x 18482427 PMC3670991

[B8] Buscemi S. Verga S. Cottone S. Azzolina V. Buscemi B. Gioia D. (2009). Glycaemic variability and inflammation in subjects with metabolic syndrome. Acta Diabetol. 46 (1), 55–61. 10.1007/s00592-008-0061-8 18818862

[B9] Chittrakarn S. Sawangjaroen K. Prasettho S. Janchawee B. Keawpradub N. (2008). Inhibitory effects of kratom leaf extract (Mitragyna speciosa Korth.) on the rat gastrointestinal tract. J. Ethnopharmacol. 116 (1), 173–178. 10.1016/j.jep.2007.11.032 18191353

[B10] Czyzyk T. A. Nogueiras R. Lockwood J. F. McKinzie J. H. Coskun T. Pintar J. E. (2010). kappa-Opioid receptors control the metabolic response to a high-energy diet in mice. Faseb J. 24 (4), 1151–1159. 10.1096/fj.09-143610 19917675 PMC2845433

[B11] Fauzi N. A. M. Tan M. L. Hamid S. B. S. Singh D. Abdullah M. F. I. L. B. (2022). Regular kratom (mitragyna speciosa korth.) use and its association with endoplasmic reticulum stress response. J. Addict. Med. 16 (6), e374–e381. 10.1097/adm.0000000000000988 35220333

[B12] Gong F. Gu H.-p. Xu Q.-t. Kang W.-y. (2012). Genus Mitragyna: ethnomedicinal uses and pharmacological studies. Phytopharmacology 3 (2), 263–272.

[B13] Grundy S. M. Cleeman J. I. Daniels S. R. Donato K. A. Eckel R. H. Franklin B. A. (2005). Diagnosis and management of the metabolic syndrome: an American heart association/national heart, lung, and blood Institute scientific statement. Circulation 112 (17), 2735–2752. 10.1161/CIRCULATIONAHA.105.169404 16157765

[B14] Hassan Z. Muzaimi M. Navaratnam V. Yusoff N. H. M. Suhaimi F. W. Vadivelu R. (2013). From Kratom to mitragynine and its derivatives: physiological and behavioural effects related to use, abuse, and addiction. Neurosci. and Biobehav. Rev. 37 (2), 138–151. 10.1016/j.neubiorev.2012.11.012 23206666

[B15] Higgins J. (2008). Cochrane handbook for systematic reviews of interventions. Cochrane Collaboration and John Wiley and Sons Ltd.

[B16] Khoirul Rista A. Ronny L. Mas Rizky Anggun Adipurna S. Kelana Kusuma D. (2022). Potential role of mitragynine as lipolysis stimulator via adrenergic signalling: docking model study. Pharmacogn. J. 14 (5), 527–531. 10.5530/pj.2022.14.130

[B17] Kunstler B. E. Cook J. L. Freene N. Finch C. F. Kemp J. L. O'Halloran P. D. (2018). Physiotherapist-led physical activity interventions are efficacious at increasing physical activity levels: a systematic review and meta-analysis. Clin. J. Sport Med. 28 (3), 304–315. 10.1097/jsm.0000000000000447 29064864

[B18] La-up A. Saengow U. Aramrattana A. (2021). High serum high-density lipoprotein and low serum triglycerides in Kratom users: a study of Kratom users in Thailand. HELIYON 7 (4), e06931. 10.1016/j.heliyon.2021.e06931 33997428 PMC8102425

[B19] La-Up A. Wongrith P. Chaichan W. Aramrattana A. Saengow U. (2022). Association between kratom (Mitragyna speciosa) use and metabolic syndrome. HELIYON 8 (5), e09468. 10.1016/j.heliyon.2022.e09468 35615431 PMC9124704

[B20] Leong Bin Abdullah M. F. I. Singh D. (2021). The adverse cardiovascular effects and cardiotoxicity of kratom (mitragyna speciosa korth.): a comprehensive review. Front. Pharmacol. 12, 726003. 10.3389/fphar.2021.726003 34646135 PMC8504575

[B21] Levesque J. Lamarche B. (2008). The metabolic syndrome: definitions, prevalence and management. J. Nutrigenetics Nutrigenomics 1 (3), 100–108. 10.1159/000112457 19776619

[B22] Modesti P. A. Reboldi G. Cappuccio F. P. Agyemang C. Remuzzi G. Rapi S. (2016). Panethnic differences in blood pressure in europe: a systematic review and meta-analysis. PLoS ONE 11 (1), e0147601. 10.1371/journal.pone.0147601 26808317 PMC4725677

[B23] Moher D. Liberati A. Tetzlaff J. Altman D. G. The P. G. (2009). Preferred reporting Items for systematic reviews and meta-analyses: the PRISMA statement. PLOS Med. 6 (7), e1000097. 10.1371/journal.pmed.1000097 19621072 PMC2707599

[B24] Musa Obadia P. Kalenda Mulaji G. Muta Musambo T. Pyana Kitenge J. Carsi Kuhangana T. Kayembe-Kitenge T. (2024). (197) natural aphrodisiacs consumption by male workers in the katanga province, DR Congo. J. Sex. Med. 21 (Suppl. ment_2). 10.1093/jsxmed/qdae002.174

[B25] National Heart L. Blood Institute (2021). Study quality assessment tools United States: United States department of health and human services. Available online at: https://www.nhlbi.nih.gov/health-topics/study-quality-assessment-tools.

[B26] O'Neill S. O'Driscoll L. (2015). Metabolic syndrome: a closer look at the growing epidemic and its associated pathologies. Obes. Rev. 16 (1), 1–12. 10.1111/obr.12229 25407540

[B27] Page M. J. McKenzie J. E. Bossuyt P. M. Boutron I. Hoffmann T. C. Mulrow C. D. (2021). The PRISMA 2020 statement: an updated guideline for reporting systematic reviews. BMJ 372, n71. 10.1136/bmj.n71 33782057 PMC8005924

[B28] Prozialeck W. C. Avery B. A. Boyer E. W. Grundmann O. Henningfield J. E. Kruegel A. C. (2019). Kratom policy: the challenge of balancing therapeutic potential with public safety. Int. J. Drug Policy 70, 70–77. 10.1016/j.drugpo.2019.05.003 31103778 PMC7881941

[B29] Rahimi N. Gozashti M. H. Najafipour H. Shokoohi M. Marefati H. (2014). Potential effect of opium consumption on controlling diabetes and some cardiovascular risk factors in diabetic patients. Addict. Health 6 (1-2), 1–6. 25140211 PMC4137437

[B30] Ramachandram D. S. Chia Siang K. Rini R. (2023). Comparison of biochemical and safety parameters of regular kratom (Mitragyna speciosa Korth.) users at two different time periods. J. Subst. Use 28 (1), 20–25. 10.1080/14659891.2021.1999513

[B31] Rana J. S. Nieuwdorp M. Jukema J. W. Kastelein J. J. P. (2007). Cardiovascular metabolic syndrome – an interplay of, obesity, inflammation, diabetes and coronary heart disease. Diabetes, Obes. Metabolism 9 (3), 218–232. 10.1111/j.1463-1326.2006.00594.x 17391148

[B32] Ribeiro C. M. Beserra B. T. S. Silva N. G. Lima C. L. Rocha P. R. S. Coelho M. S. (2020). Exposure to endocrine-disrupting chemicals and anthropometric measures of obesity: a systematic review and meta-analysis. BMJ Open 10 (6), e033509. 10.1136/bmjopen-2019-033509 PMC7311014 32565448

[B33] Saingam D. Singh D. Geater A. F. Assanangkornchai S. Jitpiboon W. Latkin C. (2023). The health impact of long-term kratom (mitragyna speciosa) use in southern Thailand. Subst. Use and Misuse 58 (10), 1212–1225. 10.1080/10826084.2023.2215301 37270449

[B34] Samson S. L. Garber A. J. (2014). Metabolic syndrome. Endocrinol. Metabolism Clin. N. Am. 43 (1), 1–23. 10.1016/j.ecl.2013.09.009 24582089

[B35] Singh D. Müller C. P. Murugaiyah V. Hamid S. B. S. Vicknasingam B. K. Avery B. (2018a). Evaluating the hematological and clinical-chemistry parameters of kratom (Mitragyna speciosa) users in Malaysia. J. Ethnopharmacol. 214, 197–206. 10.1016/j.jep.2017.12.017 29248450

[B36] Singh D. Murugaiyah V. Hamid S. B. S. Kasinather V. Chan M. S. A. Ho E. T. W. (2018b). Assessment of gonadotropins and testosterone hormone levels in regular Mitragyna speciosa (Korth.) users. J. Ethnopharmacol. 221, 30–36. 10.1016/j.jep.2018.04.005 29626673

[B37] Swarup S. Ahmed I. Grigorova Y. Zeltser R. (2024). Metabolic syndrome. Available online at: https://www.ncbi.nlm.nih.gov/books/NBK459248/. 29083742

[B38] Trakulsrichai S. Sathirakul K. Auparakkitanon S. Krongvorakul J. Sueajai J. Noumjad N. (2015). Pharmacokinetics of mitragynine in man. Drug Des. Devel Ther. 9, 2421–2429. 10.2147/dddt.S79658 PMC4425236 25995615

[B39] Utar Z. Majid M. I. A. Adenan M. I. Jamil M. F. A. Lan T. M. (2011). Mitragynine inhibits the COX-2 mRNA expression and prostaglandin E_2_ production induced by lipopolysaccharide in RAW264.7 macrophage cells. J. Ethnopharmacol. 136 (1), 75–82. 10.1016/j.jep.2011.04.011 21513785

[B40] Veltri C. Grundmann O. (2019). Current perspectives on the impact of Kratom use. Subst. Abuse Rehabil. 10, 23–31. 10.2147/sar.S164261 31308789 PMC6612999

[B41] Wan X. Wang W. Liu J. Tong T. (2014). Estimating the sample mean and standard deviation from the sample size, median, range and/or interquartile range. BMC Med. Res. Methodol. 14 (1), 135. 10.1186/1471-2288-14-135 25524443 PMC4383202

[B42] Zhang P. Wei W. Zhang X. Wen C. Ovatlarnporn C. Olatunji O. J. (2023). Antidiabetic and antioxidant activities of Mitragyna speciosa (kratom) leaf extract in type 2 diabetic rats. Biomed. and Pharmacother. 162, 114689. 10.1016/j.biopha.2023.114689 37058820

