# Supplementary material for: The effects of kratom (Mitragyna speciosa) on metabolic syndrome-related parameters: a systematic review and meta-analysis
Source: Front Pharmacol. 2025 Jun 18;16:1587528. doi: 10.3389/fphar.2025.1587528 (PMC12214432; doi:10.3389/fphar.2025.1587528)
Supplement: Supplementary file 1 [file DataSheet1.pdf]

Supplementary materials

The effects of kratom (*Mitragyna speciosa*) on metabolic syndrome-related parameters: a systematic review and meta-analysis

Ajaree Rayanakorn, Pattarachon Apisitwittaya, Shaun Wen Huey Lee, Kantirat Yaja, Ratchanon Inpan, Mingkwan Na Takuathung, Nut Koonrungsesomboon

Online supplementary contents

Appendix 1 The search strings used……………………………………………………………..………..3

Figure 1.1 PRISMA flow diagram of search strategy and study selection……………..…........5

Table 1.1 Details of 5 studies excluded after full-text review………………..……..………..6

Appendix 2 Meta-analysis………………………………………………………….……….........7

Figure 2A Forest plot summary of cholesterol mean difference (MD) and 95% confidence intervals (CIs) comparing kratom users and non-kratom users ………….………………..........7

Figure 2B Forest plot summary of low-density lipoprotein cholesterol (LDL-c) mean difference (MD) and 95% confidence intervals (CIs) comparing kratom users and non-kratom users……...7

Figure 2C Forest plot summary of high-density lipoprotein cholesterol (HDL-c) mean difference (MD) and 95% confidence intervals (CIs) comparing kratom users and non-kratom users……………………………………………………………………...……………...8

Figure 2D Forest plot summary of triglyceride mean difference (MD) and 95% confidence intervals (CIs) comparing kratom users and non-kratom users……………………………....8

Figure 2E Forest plot summary of body mass index (BMI) mean difference (MD) and 95% confidence intervals (CIs) comparing kratom users and non-kratom users……………………9

Figure 2F Forest plot summary of fasting blood sugar (FBS) mean difference (MD) and 95% confidence intervals (CIs) comparing kratom users and non-kratom users……….…………9

Figure 2G Forest plot summary of body mass index (BMI) mean difference (MD) and 95% confidence intervals (CIs) comparing kratom users and non-kratom users by study population's country……………………………………………………………………....……....10

Appendix 3 Sensitivity analysis..……………………………………………………..…………....11

Table 3.1 Sensitivity analysis of total cholesterol comparing kratom users and non-kratom users ……………………………………………………………………………………......11

Table 3.2 Sensitivity analysis of LDL-c comparing kratom users and non-kratom users …….…………………………………………………………………………….……….…12

Table 3.3 Sensitivity analysis of HDL-c comparing kratom users and non-kratom users …….…………………………………………………………………………….……….…12

Table 3.4 Sensitivity analysis of triglyceride comparing kratom users and non-kratom users…….………………………………………………………………………………...13

Table 3.5 Sensitivity analysis of BMI comparing kratom users and non-kratom users …...….14

Table 3.6 Sensitivity analysis of fasting blood sugar (FBS) comparing kratom users and non-kratom users…………………………………………………..……………………….…15

References………………………………………………………………………………...........16

Appendix 1: The search strings used

PubMed

Step	Search algorithm	Items found
#4	Search: ((((kratom) OR (mitragynine)) OR (mitragyna)) OR (Mitragyna speciosa)) AND (((((((((((((metabolic syndrome) OR (metabolic)) OR (lipid level)) OR (dyslipidemia)) OR (lipid)) OR (cholesterol)) OR (triglyceride)) OR (lipoprotein)) OR (fatty-acid)) OR (FPG)) OR (fasting plasma glucose)) OR (HbA1C)) OR (RBS)) OR (random blood sugar)) Filters: Full text, Humans	117
#3	Search: ((((kratom) OR (mitragynine)) OR (mitragyna)) OR (Mitragyna speciosa)) AND (((((((((((((metabolic syndrome) OR (metabolic)) OR (lipid level)) OR (dyslipidemia)) OR (lipid)) OR (cholesterol)) OR (triglyceride)) OR (lipoprotein)) OR (fatty-acid)) OR (FPG)) OR (fasting plasma glucose)) OR (HbA1C)) OR (RBS)) OR (random blood sugar))	232
#2	Search: ((((((((((((metabolic syndrome) OR (metabolic)) OR (lipid level)) OR (dyslipidemia)) OR (lipid)) OR (cholesterol)) OR (triglyceride)) OR (lipoprotein)) OR (fatty-acid)) OR (FPG)) OR (fasting plasma glucose)) OR (HbA1C)) OR (RBS)) OR (random blood sugar)	10,065,919
#1	Search: (((("kratom") OR ("mitragynine")) OR ("mitragyna")) OR ("Mitragyna speciosa"))	942

Cochrane

Step	Search algorithm	Items found
#4	Search: ((((kratom) OR (mitragynine)) OR (mitragyna)) OR (Mitragyna speciosa)) AND (((((((((((((metabolic syndrome) OR (metabolic)) OR (lipid level)) OR (dyslipidemia)) OR (lipid)) OR (cholesterol)) OR (triglyceride)) OR (lipoprotein)) OR (fatty-acid)) OR (FPG)) OR (fasting plasma glucose)) OR (HbA1C)) OR (RBS)) OR (random blood sugar)) Filters: Humans	0
#3	Search: ((((kratom) OR (mitragynine)) OR (mitragyna)) OR (Mitragyna speciosa)) AND (((((((((((((metabolic syndrome) OR (metabolic)) OR (lipid level)) OR (dyslipidemia)) OR (lipid)) OR (cholesterol)) OR (triglyceride)) OR (lipoprotein)) OR (fatty-acid)) OR (FPG)) OR (fasting plasma glucose)) OR (HbA1C)) OR (RBS)) OR (random blood sugar))	0
#2	Search: ((((((((((((metabolic syndrome) OR (metabolic)) OR (lipid level)) OR (dyslipidemia)) OR (lipid)) OR (cholesterol)) OR (triglyceride)) OR (lipoprotein)) OR (fatty-acid)) OR (FPG)) OR (fasting plasma glucose)) OR (HbA1C)) OR (RBS)) OR (random blood sugar)	146,553
#1	Search: (((("kratom") OR ("mitragynine")) OR ("mitragyna")) OR ("Mitragyna speciosa"))	6

Web of Science

Step	Search algorithm	Items found
#2	Search: (ALL=(“kratom” OR “mitragynine” OR “mitragyna” OR “Mitragyna speciosa”)) Filter:metabolic	29
#1	(ALL=(“kratom” OR “mitragynine” OR “mitragyna” OR “Mitragyna speciosa”))	1397

Embase

Step	Search algorithm	Items found
#4	Search: ((((kratom) OR (mitragynine)) OR (mitragyna)) OR (Mitragyna speciosa)) AND (((((((((((((metabolic syndrome) OR (metabolic)) OR (lipid level)) OR (dyslipidemia)) OR (lipid)) OR (cholesterol)) OR (triglyceride)) OR (lipoprotein)) OR (fatty-acid)) OR (FPG)) OR (fasting plasma glucose)) OR (HbA1C)) OR (RBS)) OR (random blood sugar)) Filters: Humans	48
#3	Search: ((((kratom) OR (mitragynine)) OR (mitragyna)) OR (Mitragyna speciosa)) AND (((((((((((((metabolic syndrome) OR (metabolic)) OR (lipid level)) OR (dyslipidemia)) OR (lipid)) OR (cholesterol)) OR (triglyceride)) OR (lipoprotein)) OR (fatty-acid)) OR (FPG)) OR (fasting plasma glucose)) OR (HbA1C)) OR (RBS)) OR (random blood sugar))	85
#2	Search: ((((((((((((metabolic syndrome) OR (metabolic)) OR (lipid level)) OR (dyslipidemia)) OR (lipid)) OR (cholesterol)) OR (triglyceride)) OR (lipoprotein)) OR (fatty-acid)) OR (FPG)) OR (fasting plasma glucose)) OR (HbA1C)) OR (RBS)) OR (random blood sugar)	2,712,084
#1	Search: (((("kratom") OR ("mitragynine")) OR ("mitragyna")) OR ("Mitragyna speciosa"))	1,484

Figure 1.1 PRISMA flow diagram of search strategy and study selection

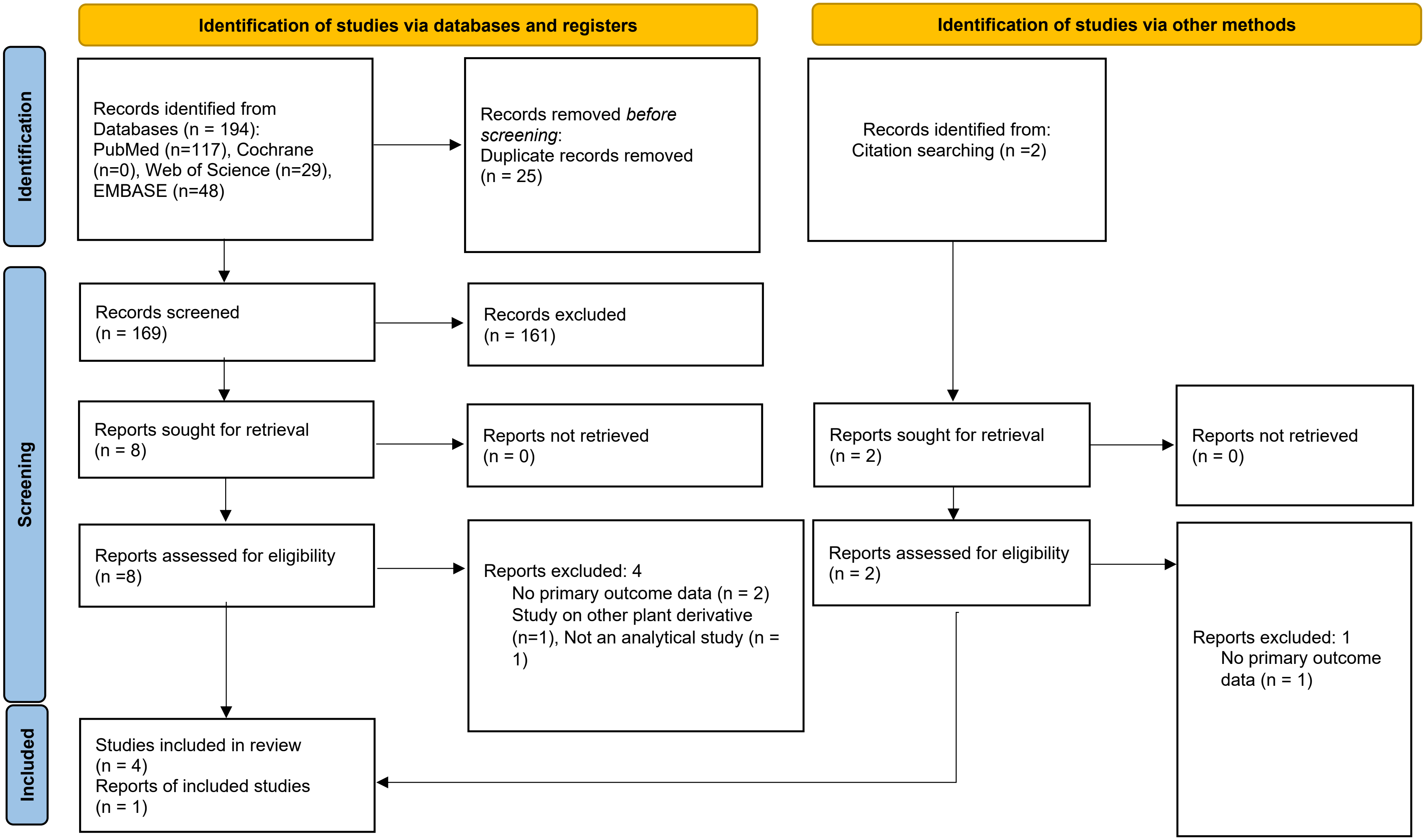

eTable 1.1 Details of 5 studies excluded after full-text review

Author	Title	Reason for exclusion
Ahmad, K & Aziz, Z.2012	Mitragyna speciosa use in the northern states of Malaysia: A cross-sectional study	No primary outcome
Fauzi NAM., et al.2022	Regular Kratom (Mitragyna speciosa Korth.) Use and Its Association With Endoplasmic Reticulum Stress Response	No primary outcome
Musa Obadia, P., et al.2024	Natural aphrodisiacs consumption by male workers in the Katanga province, Dr. Congo	Use other illicit (5 locally plant-derived aphrodisiacs): Zingiber officinale, Mitragyna stipulosa, Ocimum sp, Piper nigrum, Securidaca longepedunculata
Ramachandram, DS et al. 2023	Comparison of biochemical and safety parameters of regular kratom (Mitragyna speciosa Korth.) users at two different time periods	Not an analytical study
Saingam, D, et al.2023	The Health Impact of Long-Term Kratom (Mitragyna Speciosa) Use in Southern Thailand	No primary outcome

Appendix 2: Meta-analysis

Figure 2A Forest plot summary of total cholesterol mean difference (MD) and 95% confidence intervals (CIs) comparing kratom users and non-kratom users

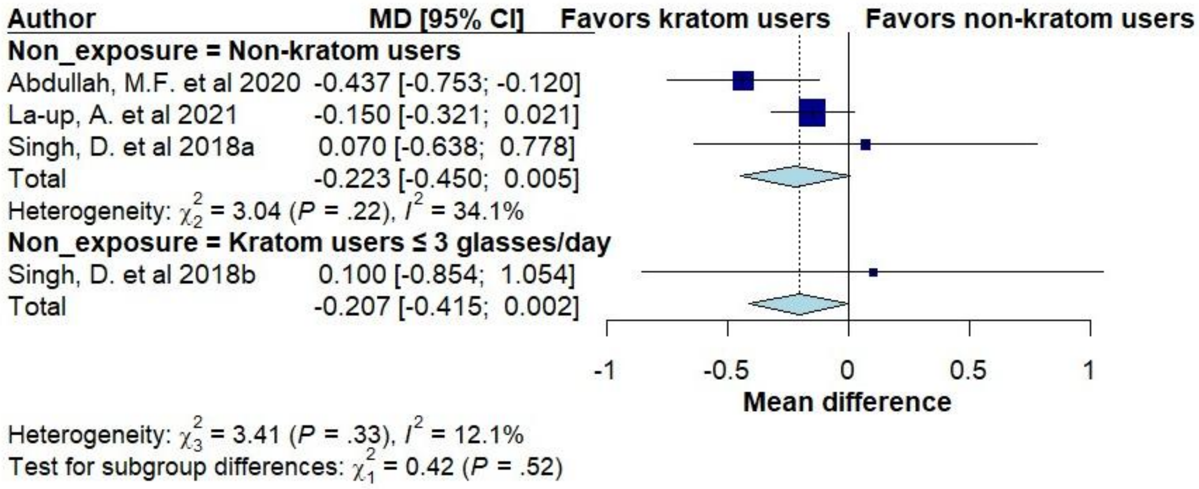

Figure 2B Forest plot summary of low-density lipoprotein cholesterol (LDL-c) mean difference (MD) and 95% confidence intervals (CIs) comparing kratom users and non-kratom users

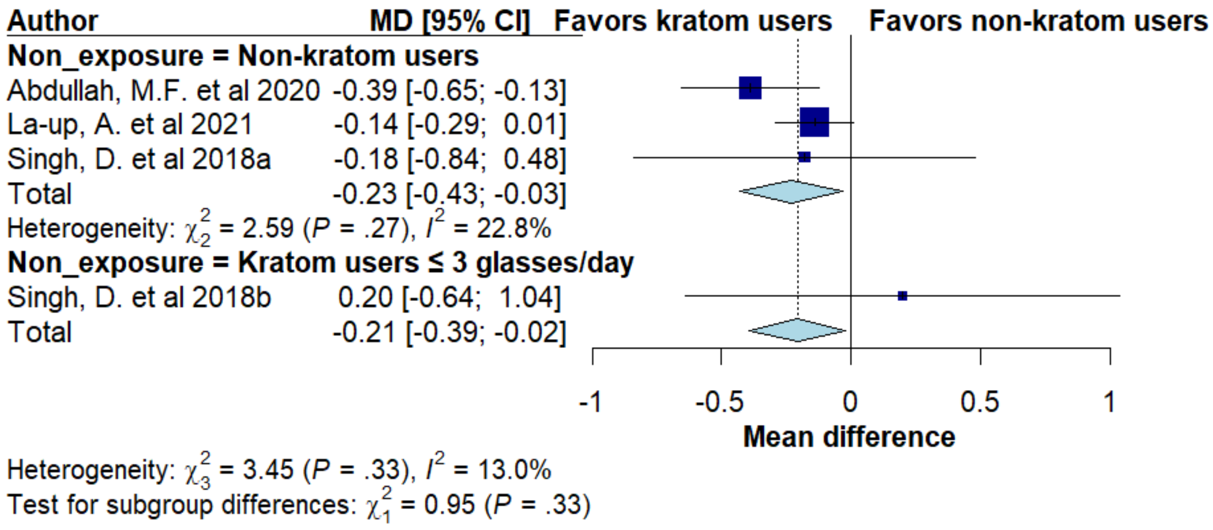

Figure 2C Forest plot summary of high-density lipoprotein cholesterol (HDL-c) mean difference (MD) and 95% confidence intervals (CIs) comparing kratom users and non-kratom users

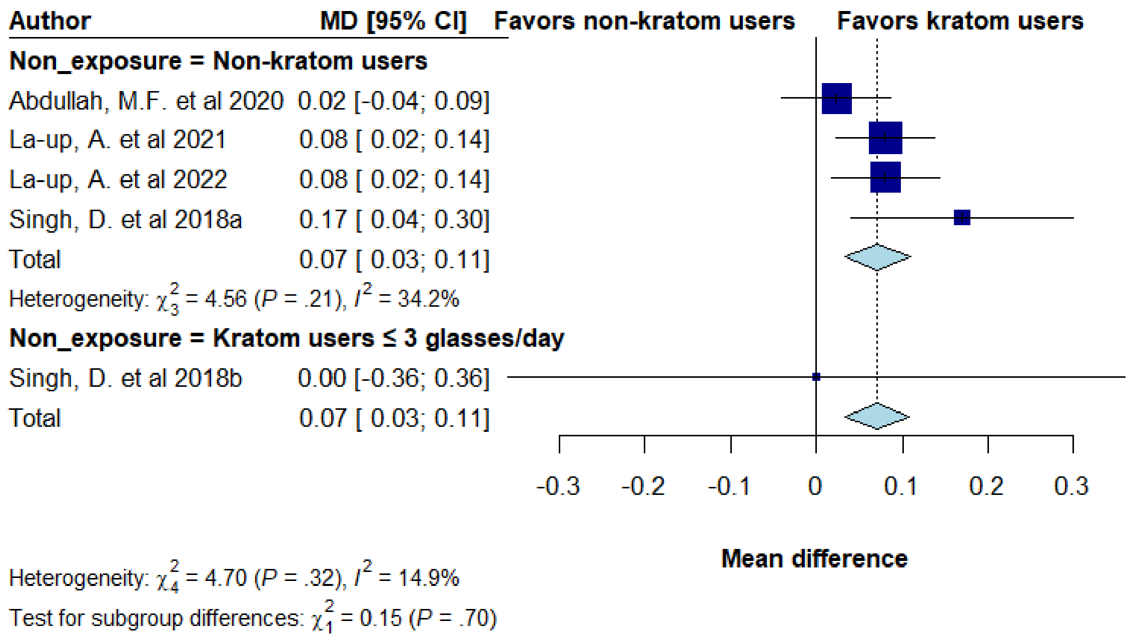

Figure 2D Forest plot summary of triglyceride mean difference (MD) and 95% confidence intervals (CIs) comparing kratom users and non-kratom users

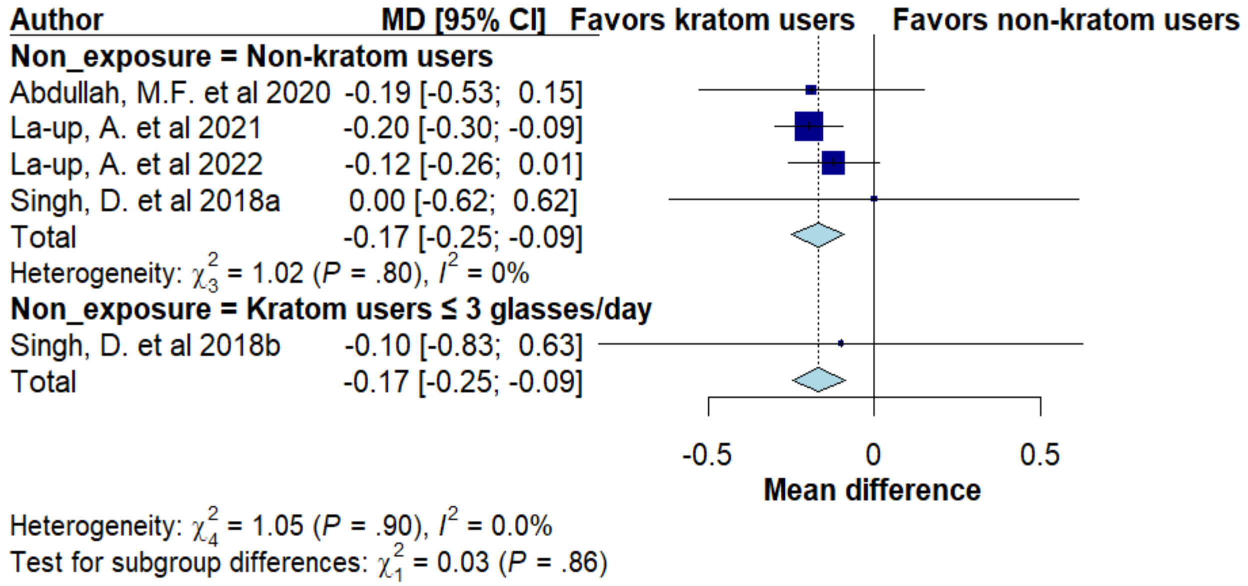

Figure 2E Forest plot summary of body mass index (BMI) mean difference (MD) and 95% confidence intervals (CIs) comparing kratom users and non-kratom users

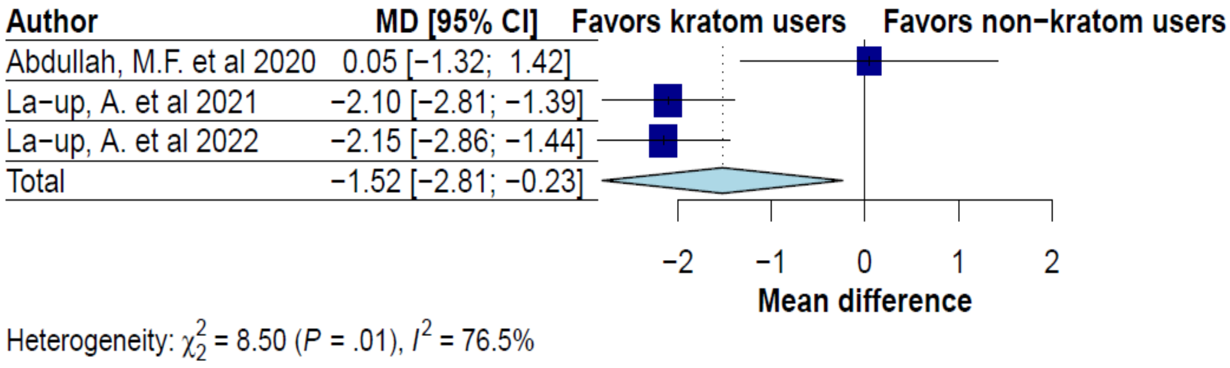

Figure 2F Forest plot summary of fasting blood sugar (FBS) mean difference (MD) and 95% confidence intervals (CIs) comparing kratom users and non-kratom users

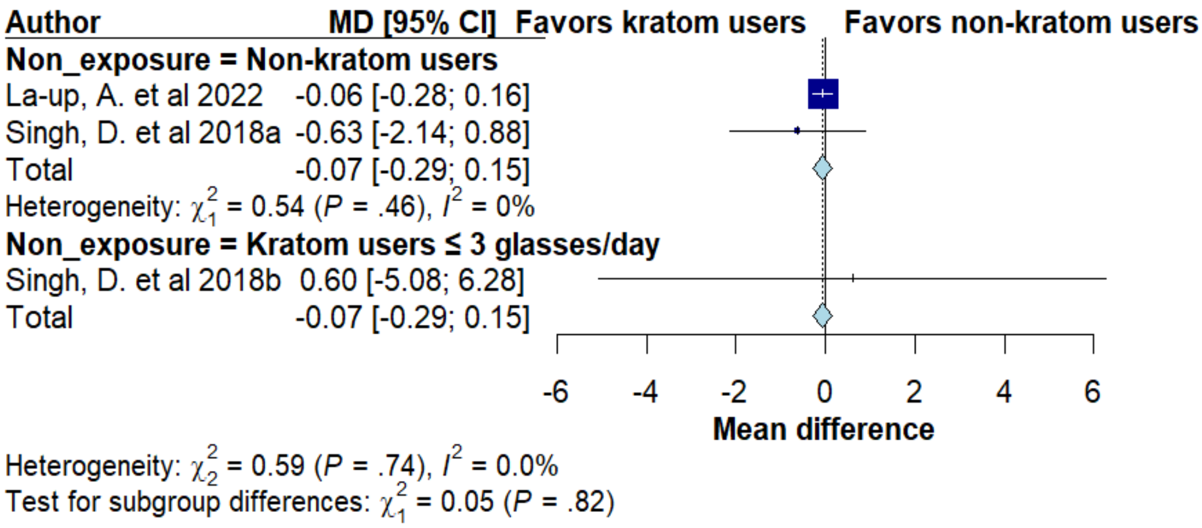

Figure 2G Forest plot summary of body mass index (BMI) mean difference (MD) and 95% confidence intervals (CIs) comparing kratom users and non-kratom users by study population's country

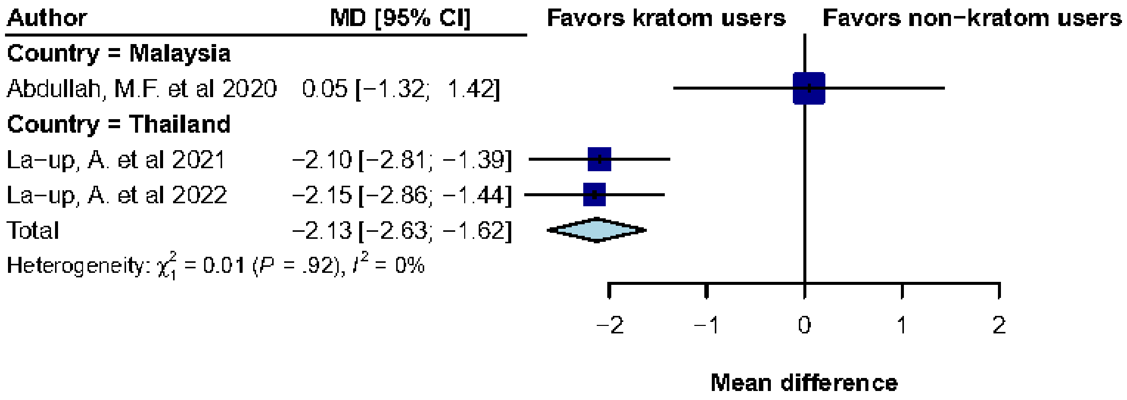

Appendix 3 Sensitivity analysis

Table 3.1 Sensitivity analysis of total cholesterol comparing kratom users and non-kratom users

Outcome	Analytic method	Number of studies	Number of participants		Heterogeneity		Effect estimates		
			Kratom users	Non-kratom users	Chi^2	I^2 (%)	MD	95% CI	*P*-value
**Total cholesterol**	Main analysis (REML)	4	452	425	0.3321	12.1	-0.2066	-0.4149 to 0.0018	0.052
	Fixed-effect estimate	4	452	425	-	-	-0.1957	-0.3413 to -0.0500	0.0085
	Paule-Mandel	4	452	425	0.3321	12.1	-0.2025	-0.3734 to -0.0315	0.0203
	DerSimonian-Laird	4	452	425	0.3321	12.1	-0.2039	-0.3823 to -0.0254	0.0251
	Maximum-likelihood	4	452	425	0.3321	12.1	-0.1957	-0.3413 to -0.0500	0.0085
	Hunter-Schmidt	4	452	425	0.3321	12.1	-0.1957	-0.3413 to -0.0500	0.0085
	Sidik-Jonkman	4	452	425	0.3321	12.1	-0.2042	-0.4529 to 0.0446	0.1077
	Hedges	4	452	425	0.3321	12.1	-0.1957	-0.3413 to -0.0500	0.0085
	Empirical Bayes	4	452	425	0.3321	12.1	-0.2025	-0.3735 to -0.0315	0.0203

Table 3.2 Sensitivity analysis of LDL-c comparing kratom users and non-kratom users

Outcome	Analytic method	Number of studies	Number of participants		Heterogeneity		Effect estimates		
			Kratom users	Non-kratom users	Chi^2	I^2 (%)	MD	95% CI	*P*-value
**LDL**	Main analysis (REML)	4	452	425	0.3276	13	-0.2068	-0.3929 to -0.0207	0.0294
	Fixed-effect estimate	4	452	425	-	-	-0.1917	-0.3190 to -0.0643	0.0032
	Paule-Mandel	4	452	425	0.3276	13	-0.1993	-0.3468 to -0.0517	0.0081
	DerSimonian-Laird	4	452	425	0.3276	13	-0.2019	-0.3588 to -0.0450	0.0117
	Maximum-likelihood	4	452	425	0.3276	13	-0.1917	-0.3190 to -0.0643	0.0032
	Hunter-Schmidt	4	452	425	0.3276	13	-0.1917	-0.3190 to -0.0643	0.0032
	Sidik-Jonkman	4	452	425	0.3276	13	-0.2071	-0.4307 to 0.0165	0.0695
	Hedges	4	452	425	0.3276	13	-0.1917	-0.3190 to -0.0643	0.0032
	Empirical Bayes	4	452	425	0.3276	13	-0.1993	-0.3468 to -0.0517	0.0081

Table 3.3 Sensitivity analysis of HDL-c comparing kratom users and non-kratom users

Outcome	Analytic method	Number of studies	Number of participants		Heterogeneity		Effect estimates		
			Kratom users	Non-kratom users	Chi^2	I^2 (%)	MD	95% CI	*P*-value
**HDL**	Main analysis (REML)	5	737	721	0.3195	14.9	0.0701	0.0329 to 0.1073	0.0002
	Fixed-effect estimate	5	737	721	-	-	0.0694	0.0355 to 0.1033	< 0.0001
	Paule-Mandel	5	737	721	0.3195	14.9	0.0704	0.0319 to 0.1090	0.0003
	DerSimonian-Laird	5	737	721	0.3195	14.9	0.0704	0.0320 to 0.1088	0.0003
	Maximum-likelihood	5	737	721	0.3195	14.9	0.0694	0.0355 to 0.1033	< 0.0001
	Hunter-Schmidt	5	737	721	0.3195	14.9	0.0694	0.0355 to 0.1033	< 0.0001
	Sidik-Jonkman	5	737	721	0.3195	14.9	0.0738	0.0202 to 0.1273	0.007
	Hedges	5	737	721	0.3195	14.9	0.0694	0.0355 to 0.1033	< 0.0001
	Empirical Bayes	5	737	721	0.3195	14.9	0.0705	0.0317 to 0.1093	0.0004
	Excluding study with high RoB	4	452	425	0.2082	34.0	0.0708	0.0098 to 0.1318	0.0229

Table 3.4 Sensitivity analysis of triglyceride comparing kratom users and non-kratom users

Outcome	Analytic method	No. of studies	Number of participants		Heterogeneity		Effect estimates		
			Kratom users	Non-kratom users	Chi^2	I^2 (%)	MD	95%CI	*P*-value
**Triglyceride**	Main analysis (REML)	5	737	721	0.9023	0	-0.1674	-0.2466 to -0.0882	< 0.0001
	Fixed-effect estimate	5	737	721	-	-	-0.1674	-0.2466 to -0.0882	< 0.0001
	Paule-Mandel	5	737	721	0.9023	0	-0.1674	-0.2466 to -0.0882	< 0.0001
	DerSimonian-Laird	5	737	721	0.9023	0	-0.1674	-0.2466 to -0.0882	< 0.0001
	Maximum-likelihood	5	737	721	0.9023	0	-0.1674	-0.2466 to -0.0882	< 0.0001
	Hunter-Schmidt	5	737	721	0.9023	0	-0.1674	-0.2466 to -0.0882	< 0.0001
	Sidik-Jonkman	5	737	721	0.9023	0	-0.1654	-0.2525 to -0.0783	< 0.0001
	Hedges	5	737	721	0.9023	0	-0.1674	-0.2466 to -0.0882	< 0.0001
	Empirical Bayes	5	737	721	0.9023	0	-0.1674	-0.2466 to -0.0882	< 0.0001
	Excluding study with high RoB	4	452	425	0.9321	0	-0.1898	-0.2870 to -0.0927	0.0001

Table 3.5 Sensitivity analysis of BMI comparing kratom users and non-kratom users

Outcome	Analytic method	Number of studies	Number of participants		Heterogeneity		Effect estimates		
			Kratom users	Non-kratom users	Chi^2	I^2 (%)	MD	95% CI	*P*-value
**BMI**	Main analysis (REML)	3	670	692	0.0142	76.5	-1.5216	-2.8083 to -0.2349	0.0205
	Fixed-effect estimate	3	670	692	-	-	-1.8696	-2.3405 to -1.3986	< 0.0001
	Paule-Mandel	3	670	692	0.0142	76.5	-1.5105	-2.8680 to -0.1530	0.0292
	DerSimonian-Laird	3	670	692	0.0142	76.5	-1.5742	-2.6184 to -0.5301	0.0031
	Maximum-likelihood	3	670	692	0.0142	76.5	-1.6015	-2.5561 to -0.6468	0.001
	Hunter-Schmidt	3	670	692	0.0142	76.5	-1.653	-2.4744 to -0.8316	< 0.0001
	Sidik-Jonkman	3	670	692	0.0142	76.5	-1.5134	-2.8515 to -0.1753	0.0266
	Hedges	3	670	692	0.0142	76.5	-1.5027	-2.9162 to -0.0891	0.0372
	Empirical Bayes	3	670	692	0.0142	76.5	-1.5105	-2.8680 to -0.1530	0.0292
	Excluding study with high RoB	2	385	396	0.0064	86.5	-1.1088	-3.2093 to 0.9918	0.3009

Table 3.6 Sensitivity analysis of fasting blood sugar (FBS) comparing kratom users and non-kratom users

Outcome	Analytic method	Number of studies	Number of participants		Heterogeneity		Effect estimates		
			Kratom users	Non-kratom users	Chi^2	I^2 (%)	MD	95% CI	*P*-value
**FBS**	Main analysis (REML)	3	352	325	0.7438	0	-0.071	-0.2891 to 0.1471	0.5236
	Fixed-effect estimate	3	352	325	-	-	-0.071	-0.2891 to 0.1471	0.5236
	Paule-Mandel	3	352	325	0.7438	0	-0.071	-0.2891 to 0.1471	0.5236
	DerSimonian-Laird	3	352	325	0.7438	0	-0.071	-0.2891 to 0.1471	0.5236
	Maximum-likelihood	3	352	325	0.7438	0	-0.071	-0.2891 to 0.1471	0.5236
	Hunter-Schmidt	3	352	325	0.7438	0	-0.071	-0.2891 to 0.1471	0.5236
	Sidik-Jonkman	3	352	325	0.7438	0	-0.1036	-0.5567 to 0.3494	0.6539
	Hedges	3	352	325	0.7438	0	-0.071	-0.2891 to 0.1471	0.5236
	Empirical Bayes	3	352	325	0.7438	0	-0.071	-0.2891 to 0.1471	0.5236

Outcome	Analytic method	Number of studies	Number of participants		Heterogeneity		Effect estimates		
			Kratom users	Non-kratom users	Chi^2	I^2 (%)	MD	95% CI	*P*-value
	Excluding study with high RoB	2	67	29	0.6814	0	-0.549	-2.0052 to 0.9072	0.4599

Reference

[1] Ahmad, K., & Aziz, Z. (2012). Mitragyna speciosa use in the northern states of Malaysia: A cross-sectional study. *Journal of Ethnopharmacology*, 141(1), 446–450. https://doi.org/10.1016/j.jep.2012.03.009

[2] Fauzi, N. A. M., Tan, M. L., Hamid, S. B. S., Singh, D., & Leong Bin Abdullah, M. F. I. (2022). Regular Kratom (Mitragyna speciosa Korth.) Use and Its Association With Endoplasmic Reticulum Stress Response. *Journal of Addiction Medicine*, 16(6), e374–e381. https://doi.org/10.1097/ADM.0000000000000955

[3] Musa Obadia, P., Kalenda Mulaji, G., Muta Musambo, T., Pyana Kitenge, J., Carsi Kuhangana, T., Kayembe-Kitenge, T., Banza Lubaba Nkulu, C., Nemery, B., & Enzlin, P. (2024). Natural Aphrodisiacs Consumption by Male Workers in the Katanga Province, DR Congo. *The Journal of Sexual Medicine*, 21(Supplement_2), qdae002.174. https://doi.org/10.1093/jsxmed/qdae002.174

[4] Ramachandram, D. S., Chia Siang, K., & Rini, R. (2023). Comparison of biochemical and safety parameters of regular kratom (Mitragyna speciosa Korth.) users at two different time periods. *Journal of Substance Use, 28*(1), 20-25. doi:10.1080/14659891.2021.1999513

[5] Saingam, D., Singh, D., Geater, A. F., Assanangkornchai, S., Jitpiboon, W., & Latkin, C. A. (2023). The Health Impact of Long-Term Kratom (Mitragyna Speciosa) Use in Southern Thailand. *Substance Use & Misuse*, 58(8), 1212–1225. https://doi.org/10.1080/10826084.2023.2215301
